# Supplementary material for: Hardwiring tissue-specific AAV transduction in mice through engineered receptor expression
Source: Nat Methods. 2023 Jun 8;20(7):1070–81. doi: 10.1038/s41592-023-01896-x (PMC10333121; doi:10.1038/s41592-023-01896-x)
Supplement: Supplementary file 1 — Supplementary Figs. 1–3. [file 41592_2023_1896_MOESM1_ESM.pdf]

nature methods

Article https://doi.org/10.1038/s41592-023-01896-x

Hardwiring tissue-specific AAV transduction in mice through engineered receptor expression

In the format provided by the
authors and unedited

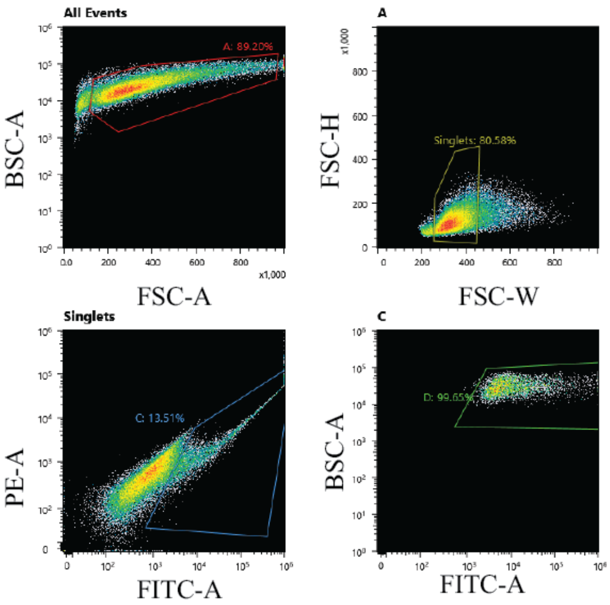

Gates and Statistics

Name	Events	%Parent	%Total
All Events	50,000	0.00%	100.00%
A	44,598	89.20%	89.20%
Singlets	35,938	80.58%	71.88%
C	4,856	13.51%	9.71%
D	4,839	99.65%	9.68%

Supplementary Figure 1: Gating strategy for transduction experiments FACS. a, MEFs transduced with AAV9-sgRNA-PCSK9-GFP were sorted to isolate the GFP+ population. FSC and SSC were used to identify single cells and GFP+ cells were gated based on a comparison to untransduced cells.

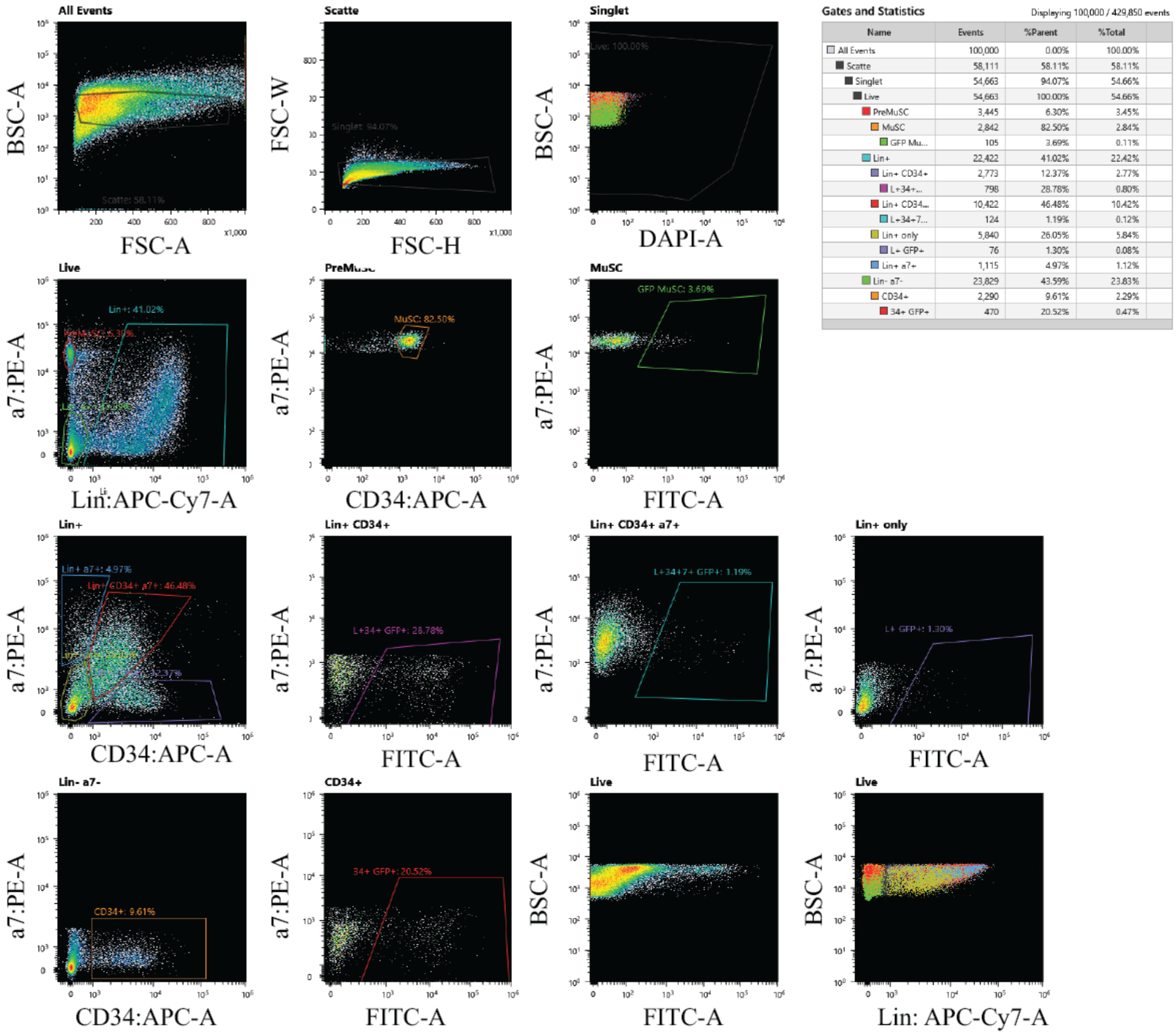

Name	Events	%Parent	%Total
All Events	100,000	0.00%	100.00%
Scatte	58,111	58.11%	58.11%
Singlet	54,663	94.07%	54.66%
Live	54,663	100.00%	54.66%
PreMuSC	3,445	6.30%	3.45%
MuSC	2,842	82.50%	2.84%
GFP Mu...	105	3.69%	0.11%
Lin+	22,422	41.02%	22.42%
Lin+ CD34+	2,773	12.37%	2.77%
L+34+...	798	28.78%	0.80%
Lin+ CD34...	10,422	46.48%	10.42%
L+34+7...	124	1.19%	0.12%
Lin+ only	5,840	26.05%	5.84%
L+ GFP+	76	1.30%	0.08%
Lin+ a7+	1,115	4.97%	1.12%
Lin- a7-	23,829	43.59%	23.83%
CD34+	2,290	9.61%	2.29%
34+ GFP+	470	20.52%	0.47%

Supplementary Figure 2: MuSCs were isolated from TA muscle and diaphragm of mice and digested to get a single cell suspension. Live mononuclear cells dissociated from AAV injected muscles were gated by FSC-A vs. SSC-A to remove low and high SSC debris. Then singlets were selected for by gating on FSC-w and FSC-h. Live cells were selected based on low DAPI signal. MuSCs were enriched by gating for a7-PE positivity and lineage marker (CD45-PE-Cy7, CD11b-PE-Cy7, CD31-PE-Cy7) negativity and further selected by gating on CD34-APC positive and SSC-A low populations. Based on previous data, this strategy yields MuSCs at >95% purity based on Pax7+ staining. Antibody gates and compensation were established using unstained, single stained, and FMO controls. GFP gates were established on untransduced cells or tissues.

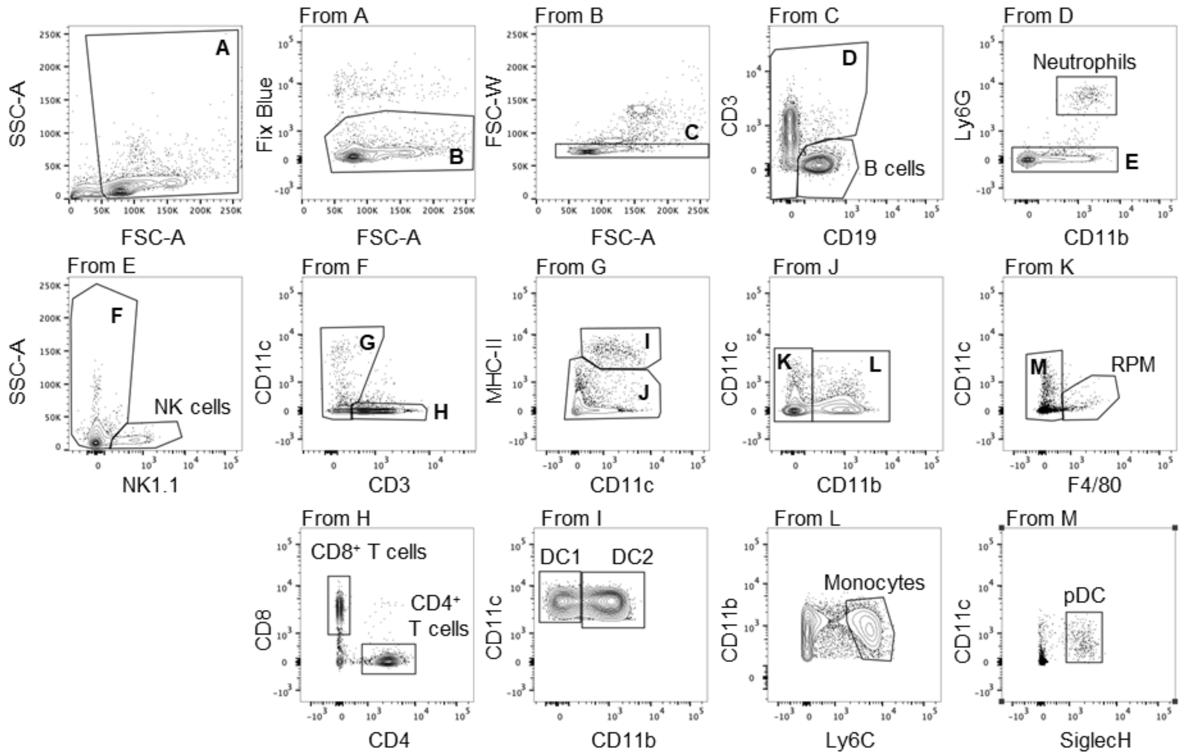

Supplementary Figure 3: Gating strategy for immune cell identification using FACS. Live cells were gated from the Fix Blue negative population, followed by singlet gating. Cell types were identified as outlined: B-cell (CD19+, CD3-), Neutrophil (CD11b+, Ly6G+), NK cells (NK1.1+), CD8+ T cells (CD3+, CD8+), CD4+ T cells (CD3+, CD4+), DC1 (MHC-II+, CD11c+, CD11b-), DC2 (MHC-II+, CD11c+, CD11b+), Monocytes (CD11b+, Ly6C+), RPM (CD11b-, F4/80+), pDC (F4/80-, Siglec+). Population frequency was identified and reported in Extended Data Fig. 2c. DC populations were combined to simplify reporting of immune cell phenotype.
