# Supplementary material for: Hardwiring tissue-specific AAV transduction in mice through engineered receptor expression
Source: Nat Methods. 2023 Jun 8;20(7):1070–81. doi: 10.1038/s41592-023-01896-x (PMC10333121; doi:10.1038/s41592-023-01896-x)
Supplement: Supplementary file 2 — Reporting Summary [file 41592_2023_1896_MOESM2_ESM.pdf]

nature research

Corresponding author(s): Jan Carette

Last updated by author(s): Mar 7, 2023

Reporting Summary

Nature Research wishes to improve the reproducibility of the work that we publish. This form provides structure for consistency and transparency in reporting. For further information on Nature Research policies, see our Editorial Policies and the Editorial Policy Checklist.

Statistics

For all statistical analyses, confirm that the following items are present in the figure legend, table legend, main text, or Methods section.

n/a	Confirmed	
☐	☒	The exact sample size (*n*) for each experimental group/condition, given as a discrete number and unit of measurement
☐	☒	A statement on whether measurements were taken from distinct samples or whether the same sample was measured repeatedly
☐	☒	The statistical test(s) used AND whether they are one- or two-sided<br>*Only common tests should be described solely by name; describe more complex techniques in the Methods section.*
☐	☒	A description of all covariates tested
☐	☒	A description of any assumptions or corrections, such as tests of normality and adjustment for multiple comparisons
☐	☒	A full description of the statistical parameters including central tendency (e.g. means) or other basic estimates (e.g. regression coefficient) AND variation (e.g. standard deviation) or associated estimates of uncertainty (e.g. confidence intervals)
☐	☒	For null hypothesis testing, the test statistic (e.g. *F*, *t*, *r*) with confidence intervals, effect sizes, degrees of freedom and *P* value noted<br>*Give P values as exact values whenever suitable.*
☒	☐	For Bayesian analysis, information on the choice of priors and Markov chain Monte Carlo settings
☒	☐	For hierarchical and complex designs, identification of the appropriate level for tests and full reporting of outcomes
☒	☐	Estimates of effect sizes (e.g. Cohen's *d*, Pearson's *r*), indicating how they were calculated

Our web collection on statistics for biologists contains articles on many of the points above.

Software and code

Policy information about availability of computer code

Data collection	In vivo luciferase data was collected using Aura version 4.0.0 (Spectral Instruments Imaging). Luciferase data was collected using GloMax (Promega). Retinal imaging was collected using Heidelberg Spectralis SLO/OCT system software. FACS data was collected using SH800S (Sony) and FACSDiva version 8.0.1 (BD). LAS X, Echo Revolve D270 version 3.0.3, Keyence BZ-X800 software, and Zeiss Zen software were used for image collection.
Data analysis	In vivo luciferase data was analyzed using Aura version 4.0.0 (Spectral Instruments Imaging). GraphPad Prism 8.0.2 was use for data analysis and statistical testing. LAS X 3.7.4.23463, ImageJ version 1.52n, Zeiss Zen black 2.3 SP1 for imaging z-stack and max projections and Zeiss Zen Blue 2.3 for quantification, and Keyence BZ-X800 software version 1.1.2.4 were used for fluorescent image processing. Sequencher version 5.1 was used for sequence analysis. FlowJo version 10.8.2 and Sony Cell Sorter software version 2.1.5 was used for FACS analysis. Western Blot analysis was performed using Bio-Rad Image Lab Software version 6.1.0 build 7.

For manuscripts utilizing custom algorithms or software that are central to the research but not yet described in published literature, software must be made available to editors and reviewers. We strongly encourage code deposition in a community repository (e.g. GitHub). See the Nature Research guidelines for submitting code & software for further information.

Data

Policy information about availability of data

All manuscripts must include a data availability statement. This statement should provide the following information, where applicable:

- Accession codes, unique identifiers, or web links for publicly available datasets
- A list of figures that have associated raw data
- A description of any restrictions on data availability

Statistical source data is included for Fig. 1, 2, 3, 4, and 5 and Extended Data Fig. 2. Unprocessed Western Blots are provided for Extended Data. Fig. 2. All other

datasets generated during and/or analysed during the current study are available from the corresponding author on reasonable request.
Chat expression in mouse brains from Extended Data Fig 9a is available through Allen Brain Atlas (https://mouse.brain-map.org/experiment/show?id=253). AAVR expression data was obtain from the Tabula Muris (https://www.czbiohub.org/sf/tabula-muris/).
Mice have been deposited with The Jackson Laboratory Repository with the SELECTIV mice (JAX Stock No. 037553 C57BL/6J-Igs2tm1(CAG-AU040320/mCherry,-cas9*)Janc/J) and Aavr-KO mice backcrossed to C57BL/6 (JAX Stock No. 037596 B6.FVB-AU040320em1Janc/J).

Field-specific reporting

Please select the one below that is the best fit for your research. If you are not sure, read the appropriate sections before making your selection.

☒ Life sciences ☐ Behavioural & social sciences ☐ Ecological, evolutionary & environmental sciences

For a reference copy of the document with all sections, see nature.com/documents/nr-reporting-summary-flat.pdf

Life sciences study design

All studies must disclose on these points even when the disclosure is negative.

Sample size

Sample sizes were not determined before each study. The number of replicates was chosen for in vivo studies based on the number of mice of each genotype available and reasonable to use for each experiment. In vitro experiments had an n>=3 and listed in the manuscript. For most studies the number of mice that were age matched with the required genotypes were used whenever possible. This resulted in varying group sizes for some studies with n between 3 and 5 for most experiments with group size listed in the mansucript.

Data exclusions

No relevant data were excluded. Some timepoints were not collected with the in vivo imaging due to death of mice. We do not believe deaths were associated with any experimental procedures.

Replication

As no data were excluded, all values from each experiment are presented and the variation can be seen in the figures. Statistical analysis as described throughout the manuscript provided the ability to confidently assess differences between different experimental conditions.

Randomization

No randomization was used. Mice were used from litters as needed for experiments. Mice of different genotypes were not treated differently and were not

Blinding

While no intentional binding was used during AAV injections and data collection, mice and samples were number by toe tattoo and were all treated similarly. Mice of different genotypes were co-housed injections, samples processing, and data acquisition were performed in parallel without regard for genotype. When bias was possible in cell type identification in retinal transduction, data was collected and analyzed in a blinded manner as outlined in the methods.

Reporting for specific materials, systems and methods

We require information from authors about some types of materials, experimental systems and methods used in many studies. Here, indicate whether each material, system or method listed is relevant to your study. If you are not sure if a list item applies to your research, read the appropriate section before selecting a response.

Materials & experimental systems

n/a	Involved in the study	
☐	☒	Antibodies
☐	☒	Eukaryotic cell lines
☒	☐	Palaeontology and archaeology
☐	☒	Animals and other organisms
☒	☐	Human research participants
☒	☐	Clinical data
☒	☐	Dual use research of concern

Methods

n/a	Involved in the study	
☒	☐	ChIP-seq
☐	☒	Flow cytometry
☒	☐	MRI-based neuroimaging

Antibodies

Antibodies used

rabbit anti-KIAA0319L, Proteintech, 21016-1-AP, 1:1000
anti-rabbit-HRP, Genetex, GTX213110-01, 1:4000
PE-Cy7-CD11b, Biolegend, M1/70, cat# 101216, 3µl/mouse
PE-Cy7-CD45, Biolegend, 23-F11, cat# 103114, 3µl/mouse
PE-Cy7-Sca1, Biolegend, D7, cat# 108114, 3µl/mouse
PE-Cy7-CD31, Biolegend, 390 cat# 102418, 3µl/mouse
PE-a7-integrin, AbLab, R2F2, SKU: 53-0010-05, 2µl/mouse
APC-CD34, Biolegend, RAM34, cat# 128612, 3µl/mouse
RBPMS, PhosphoSolutions, cat# 1832-RBPMS, 1:2000
anti-Guinea pig Alexa Fluor 647, Invitrogen, A-21450, 1:200
anti-CD3-FITC (ThermoFisher, 11-0033-82), 1:100

anti-F4/80-PerCP-Cy5.5 (ThermoFisher, 45-4801-82), 1:100
anti-SiglecH-APC (Biolegend, 129611), 1:200
anti-Ly6G-Alexa Fluor® 700 (Biolegend, 127622), 1:100
anti-CD11c-APC-eFluor™ 780 (ThermoFisher, 47-0114-82), 1:200
anti-Ly6C-eFluor 450, eBioscience™ (ThermoFisher, 48-5932-82), 1:100
anti-MHC-II-Brilliant Violet 510™ (Biolegend, 748845), 1:200
anti-CD11b-Brilliant Violet 650™ (Biolegend, 101259), 1:400
anti-CD19-BV78Brilliant Violet 785™5 (Biolegend, 115543), 1:100
anti-FIXBlue-LIVE/DEAD™ Fixable Blue Dead Cell Stain (ThermoFisher, L23105), 1:200
anti-CD8-BUV737 (BD Bioscience, 612759), 1:400
anti-CD4-BUV395 (BD Bioscience, 563790), 1:200
anti-NK1.1-PE-Cy7 (ThermoFisher, 25-5941-82), 1:100
anti-CD16/32 mAb (clone 2.4G2; produced in house)

Validation

Rabbit anti-KIAA0319L antibody was validated on AAVR KO cells and tissue (as shown in supplemental).
anti-CD16/32 mAb was produced by hybridoma in house and tested functionally for Fc receptor blockade for mouse cells prior to use.
All other antibodies were validated by supplier prior to use as described below.
PE-Cy7-CD11b, Biolegend, M1/70, cat# 101216, Application: FC (Flow Cytometry).The validation has been performed by the manufacturer: https://www.biolegend.com/en-ie/products/pe-cyanine7-anti-mouse-human-cd11b-antibody-1921
PE-Cy7-CD45, Biolegend, 23-F11, cat# 103114, Application: FC (Flow Cytometry).The validation has been performed by the manufacturer: https://www.biolegend.com/en-us/products/pe-cyanine7-anti-mouse-cd45-antibody-1903
PE-Cy7-Sca1, Biolegend, D7, cat# 108114, Application: FC (Flow Cytometry).The validation has been performed by the manufacturer: https://www.biolegend.com/en-us/search-results/pe-cyanine7-anti-mouse-ly-6a-e-sca-1-antibody-3137
PE-Cy7-CD31, Biolegend, 390 cat# 102418, Application: FC (Flow Cytometry).The validation has been performed by the manufacturer: https://www.biolegend.com/en-us/products/pe-cyanine7-anti-mouse-cd31-antibody-3942
PE-a7-integrin, AbLab, R2F2, SKU: 53-0010-05, Application: FC (Flow Cytometry).The validation in a published protocol: https://www.ncbi.nlm.nih.gov/pmc/articles/PMC5034768/
APC-CD34, Biolegend, RAM34, cat# 128612, Application: FC (Flow Cytometry).The validation has been performed by the manufacturer: https://www.biolegend.com/en-us/products/apc-anti-mouse-cd34-antibody-6520
RBPMS, PhosphoSolutions, cat# 1832-RBPMS, Application: IFA (Flow Cytometry).The validation has been performed by the manufacturer: https://www.phosphosolutions.com/products/anti-rbpms-antibody-1832-rbpms
anti-Guinea pig Alexa Fluor 647, Invitrogen, A-21450, Application: IFA (Flow Cytometry).The validation has been performed by the manufacturer: https://www.thermofisher.com/antibody/product/Goat-anti-Guinea-Pig-IgG-H-L-Highly-Cross-Adsorbed-Secondary-Antibody-Polyclonal/A-21450
anti-CD3-FITC (ThermoFisher, 11-0033-82), Application: FC (Flow Cytometry).The validation has been performed by the manufacturer: https://www.thermofisher.com/antibody/product/CD3e-Antibody-clone-eBio500A2-500A2-Monoclonal/11-0033-82
anti-F4/80-PerCP-Cy5.5 (ThermoFisher, 45-4801-82), Application: FC (Flow Cytometry).The validation has been performed by the manufacturer: https://www.thermofisher.com/antibody/product/F4-80-Antibody-clone-BM8-Monoclonal/45-4801-82
anti-SiglecH-APC (Biolegend, 129611), Application: FC (Flow Cytometry).The validation has been performed by the manufacturer: https://www.biolegend.com/en-us/products/apc-anti-mouse-siglec-h-antibody-6906
anti-Ly6G-Alexa Fluor® 700 (Biolegend, 127622), Application: FC (Flow Cytometry).The validation has been performed by the manufacturer: https://www.biolegend.com/en-us/products/alexa-fluor-700-anti-mouse-ly-6g-antibody-6754
anti-CD11c-APC-eFluor™ 780 (ThermoFisher, 47-0114-82), Application: FC (Flow Cytometry).The validation has been performed by the manufacturer: https://www.thermofisher.com/antibody/product/CD11c-Antibody-clone-N418-Monoclonal/47-0114-82
anti-Ly6C-eFluor 450, eBioscience™ (ThermoFisher, 48-5932-82), Application: FC (Flow Cytometry).The validation has been performed by the manufacturer: https://www.thermofisher.com/antibody/product/Ly-6C-Antibody-clone-HK1-4-Monoclonal/48-5932-82
anti-MHC-II-Brilliant Violet 510™ (Biolegend, 748845), Application: FC (Flow Cytometry).The validation has been performed by the manufacturer: https://www.biolegend.com/de-de/products/brilliant-violet-510-anti-mouse-i-a-i-e-antibody-7997
anti-CD11b-Brilliant Violet 650™ (Biolegend, 101259), Application: FC (Flow Cytometry).The validation has been performed by the manufacturer: https://www.biolegend.com/en-us/products/brilliant-violet-650-anti-mouse-human-cd11b-antibody-7638
anti-CD19-BV78Brilliant Violet 785™ (Biolegend, 115543), Application: FC (Flow Cytometry).The validation has been performed by the manufacturer: https://www.biolegend.com/en-ie/products/brilliant-violet-785-anti-mouse-cd19-antibody-7962
anti-FIXBlue-LIVE/DEAD™ Fixable Blue Dead Cell Stain (ThermoFisher, L23105), Application: FC (Flow Cytometry).The validation has been performed by the manufacturer: https://www.thermofisher.com/order/catalog/product/L23105
anti-CD8-BUV737 (BD Bioscience, 612759), Application: FC (Flow Cytometry).The validation has been performed by the manufacturer: https://www.bdbiosciences.com/en-us/products/reagents/flow-cytometry-reagents/research-reagents/single-color-antibodies-ruo/buv737-rat-anti-mouse-cd8a.612759
anti-CD4-BUV395 (BD Bioscience, 563790), Application: FC (Flow Cytometry).The validation has been performed by the manufacturer: https://www.bdbiosciences.com/en-us/products/reagents/flow-cytometry-reagents/research-reagents/single-color-antibodies-ruo/buv395-rat-anti-mouse-cd4.565974
anti-NK1.1-PE-Cy7 (ThermoFisher, 25-5941-82), Application: FC (Flow Cytometry).The validation has been performed by the manufacturer: https://www.thermofisher.com/antibody/product/NK1-1-Antibody-clone-PK136-Monoclonal/25-5941-82

Eukaryotic cell lines

Policy information about cell lines

Cell line source(s)

Mouse embryonic fibroblasts (MEFS) and myoblasts were generated as described in the methods from control and SELECTIV-WB mice.

Authentication

We used primary MEFs and myoblasts using standard isolation protocols.

Mycoplasma contamination

Cell lines are negative for mycoplasma as tested by mycoalert plus mycoplasma detection kit (Lonza).

Commonly misidentified lines
(See ICLAC register)

No commonly misidentified lines were used in these studies.

Animals and other organisms

Policy information about studies involving animals; ARRIVE guidelines recommended for reporting animal research

Laboratory animals	Mouse lines purchased from Jackson Laboratory include C57BL/6J (664, Jax), E2A-Cre (B6.FVB-Tg(EIIa-cre)C5379Lmgd/J, 3724, Jax), Chat-Cre (ChAT-IRES-Cre, 6410, Jax), Alb-Cre (B6.Cg-Speer6-ps1Tg(Alb-cre)21Mgn/J, 3574, Jax), Myh6-Cre (B6.FVB-Tg(Myh6-cre)2182Mds/J HEMI, 11038, Jax), and Pax7-Cre (B6.Cg-Pax7tm1(cre/ERT2)Gaka/J, 17763, Jax). FVB AAVR KO mice were previously generated. The AAVR KO allele was introduced into C57BL/6J by backcrossing for 10 generations. SELECTIV mice were generated at the Stanford Transgenic, Knockout, and Tumor Model Center using Integrase Mediated Transgenesis61 using the construct described above and PhiC31 integrase. The construct was inserted into the H11 locus using C57BL/6 mice with three attP sites previously knocked into the H11 locus. Male and female mice were used for all studies as available using littermate or age matched controls. All mice were 6-12 weeks old at time of AAV injection, except for retinal transduction studies where mice were 5 months old.
Wild animals	This study did not involve wild animals.
Field-collected samples	This study did not involve samples collected from the field.
Ethics oversight	At Stanford University, the Institutional Animal Care and Use Committee (IACUC) is appointed by the University Vice Provost and Dean of Research, and is known as the Administrative Panel on Laboratory Animal Care (APLAC). Stanford's APLAC membership is comprised of faculty, veterinarians, public members, students, and senior staff. The APLAC reports to the Office of the Vice Provost and Dean of Research. The laboratory animal care program at Stanford is accredited by AAALAC International. All procedures were carried out under the approved protocol APLAC #28856. Mice were housed in the AAALAC-accredited Stanford mouse barrier facility. Husbandry was performed in accordance with the Guide for the Care and Use of Laboratory Animals, 8th edition (PMID 21595115), and the Public Health Service Policy on Humane Care and Use of Laboratory Animals (2015).

Note that full information on the approval of the study protocol must also be provided in the manuscript.

Flow Cytometry

Plots

Confirm that:

- [x] The axis labels state the marker and fluorochrome used (e.g. CD4-FITC).
- [x] The axis scales are clearly visible. Include numbers along axes only for bottom left plot of group (a 'group' is an analysis of identical markers).
- [x] All plots are contour plots with outliers or pseudocolor plots.
- [x] A numerical value for number of cells or percentage (with statistics) is provided.

Methodology

Sample preparation

Mouse hindlimb muscles were carefully dissected to remove adipose, tendon and nerves and minced using scissors until homogenous. Minced tissue was suspended in 10mL of collagenase solution (700U/mL collagenase II (Worthington), 0.2% BSA (Sigma) in Ham's F-10 media (Gibco) and digested at 37C for 1h using the gentleMACS Octo Dissociator. Digested tissues were diluted with 20mL of 0.2% BSA in Ham's F-10 media) and pelleted at 500g for 10mins. Supernatant removed leaving 8mL of solution and cell pellet. Cell pellets were resuspended and 1000U of collagenase II and 11U of dispase I (Life Technologies) in 2mL of PBS were added and further digested for 30mins on the gentleMACS Dissociator using a custom program. Cells were pelleted, washed in FACS buffer (0.5% BSA, 1mM EDTA in PBS) and filtered through a 40um nylon cell filter. Red blood cells were lysed using RBC Lysis Buffer (eBioscience). Single-cell suspensions were incubated with direct APC-Cy7 conjugated antibodies against CD11b (M1/70), CD45 (30-F11), Sca1 (D7), and CD31 (390) (Biolegend, 3uL of each/mouse), PE-a7-integrin (2uL/mouse, AbLab), and APC-CD34 (RAM34, Biolegend). Cell suspensions were then subjected to FACS sorting for MuSC isolation (CD45– CD11b– CD31– Sca1– α7-integrin+ CD34+ cells) or flow cytometry quantification for GFP+ MuSCs (CD45– CD11b– CD31– Sca1– α7-integrin+).

To assess the number of immune cells in the spleen, spleens were harvested and single cell suspensions were acquired by mechanical disruption. Suspensions were void of red blood cells via lysis with Ammonium-Chloride-Potassium (ACK) Lysis buffer (Lonza). Subsequently, cell suspensions were incubated with anti-CD16/32 mAb (clone 2.4G2; produced in house) for 20 minutes at 4°C to block Fc receptors. Cells were washed with PBS and stained with primary antibodies and LIVE/DEAD Fixable Blue (ThermoFisher) in PBS for 25 minutes at 4°C. Cells were then fixed with BD Cytofix/Cytoperm Fixation and Permeabilization solution (BD Bioscience) for 12 mins at 4°C and then washed and resuspended with PBS. Samples were acquired on a 5-laser LSRFortessa X-20 (BD Biosciences), and data was analyzed using FlowJo software (Tree Star, Inc). Unstained and single-fluorochrome-stained cells were used for compensation. Antibodies used were anti-CD3-FITC (ThermoFisher, 11-0033-82), anti-F4/80-PerCP-Cy5.5 (ThermoFisher, 45-4801-82), anti-SiglecH-APC (Biolegend, 129611), anti-Ly6G-Alexa Fluor® 700 (Biolegend, 127622), anti-CD11c-APC-eFluor™ 780 (ThermoFisher, 47-0114-82), anti-Ly6C-eFluor 450, eBioscience™ (ThermoFisher, 48-5932-82), anti-MHC-II-Brilliant Violet 510™ (Biolegend, 748845), anti-CD11b-Brilliant Violet 650™ (Biolegend, 101259), anti-CD19-BV78Brilliant Violet 785™5 (Biolegend, 115543), anti-FIXBlue-LIVE/DEAD™ Fixable Blue Dead Cell Stain (ThermoFisher, L23105), anti-CD8-BUV737 (BD Bioscience, 612759), anti-CD4-BUV395 (BD Bioscience, 563790) and,anti-NK1.1-PE-Cy7 (ThermoFisher, 25-5941-82).

Instrument	MEF and Muscle samples: Sony SH800S and BD LSR II UV. analyzer. Spleen samples: 5-laser LSRFortessa X-20 (BD Biosciences)
Software	Analysis was performed with FlowJo and Sony Cell Sorter Software
Cell population abundance	After processing MuSCs made up ~1-2% of cells in the diaphragm samples and 2-5% of cells in the TA samples. MEF transduction was seen by GFP positivity in ~13-14% of cells.
Gating strategy	Flow cytometry and sorting strategies for MuSCs were performed as previously described in Sacco et al., 2008 Nature, Cosgrove, et al. 2014 Nat Med, Ho et al., 2017 PNAS. Briefly, live mononuclear cells dissociated from AAV injected muscles were gated by FSC-A vs. SSC-A to remove low and high SSC debris. Then singlets were selected for by gating on FSC-w and FSC-h. Live cells were selected based on low DAPI signal. MuSCs were enriched by gating for a7-PE positivity and lineage marker (CD45-PE-Cy7, CD11b-PE-Cy7, CD31-PE-Cy7, ScaI-PE-Cy7) negativity and further selected by gating on CD34-APC positive and SSC-A low populations. Based on previous data, this strategy yields MuSCs at >95% purity based on Pax7+ staining. Antibody gates and compensation were established using unstained, single stained, and FMO controls. GFP gates were established on untransduced cells or tissues.<br><br>Gating strategy for immune cells is shown in Supplementary Fig. 3. Cells were gating on SSC-A/FSC-A, followed by isolation of live cells that did not stain positive for Fix Blue. Singlets were then isolated on a FSC-W/FSC-A plot. CD19+/CD3- cells were called B-cells. The rest of the cells were further gated, with Ly6G+ CD11b+ cells being neutrophils and the rest of the cells further gated to identify NK1.1 cells as NK cells. The rest of the cells were further gated to identify CD3+ CD8+ to be CD8+ T-cells and CD3+ CD4+ to be CD4+ T cells. CD11c+ cells were identified as dendritic cells. And Ly6c+ cells were identified as Monocytes.

☒ Tick this box to confirm that a figure exemplifying the gating strategy is provided in the Supplementary Information.
