# Supplementary material for: Hardwiring tissue-specific AAV transduction in mice through engineered receptor expression
Source: Nat Methods. 2023 Jun 8;20(7):1070–81. doi: 10.1038/s41592-023-01896-x (PMC10333121; doi:10.1038/s41592-023-01896-x)

Liver

Anti-AAVR

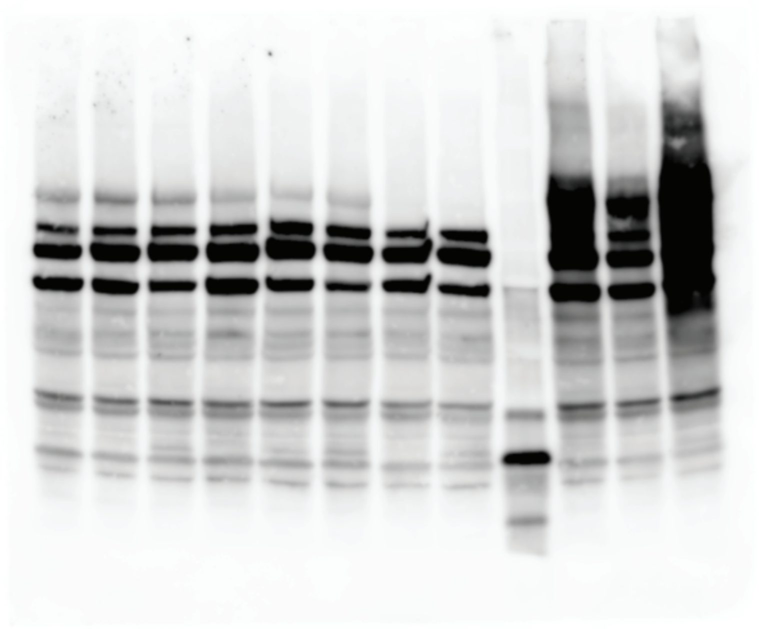

Anti-GAPDH

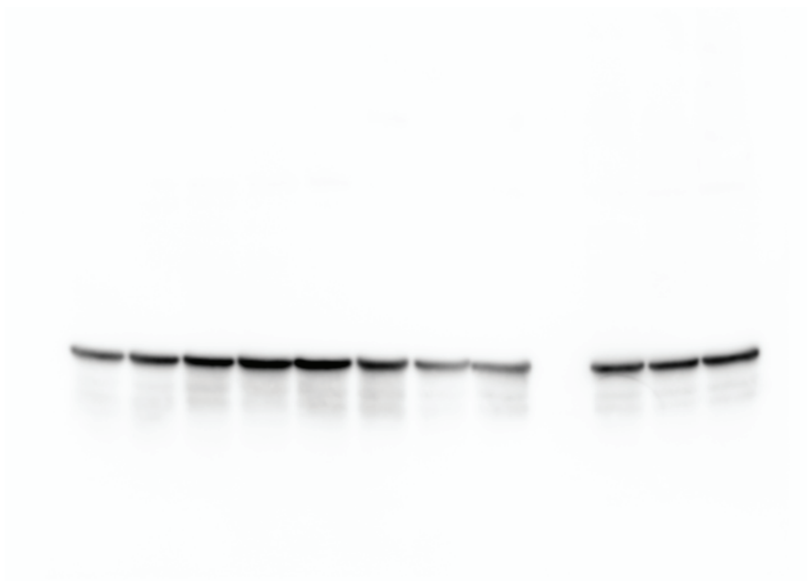

Precision Plus
Protein Dual
Color Standard

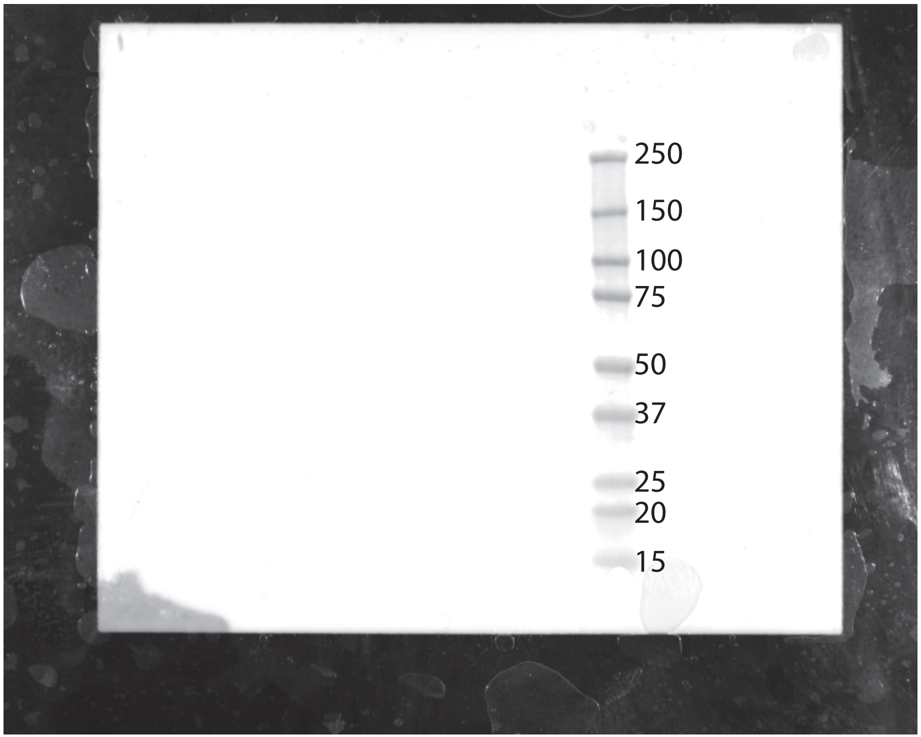

Heart

Anti-AAVR

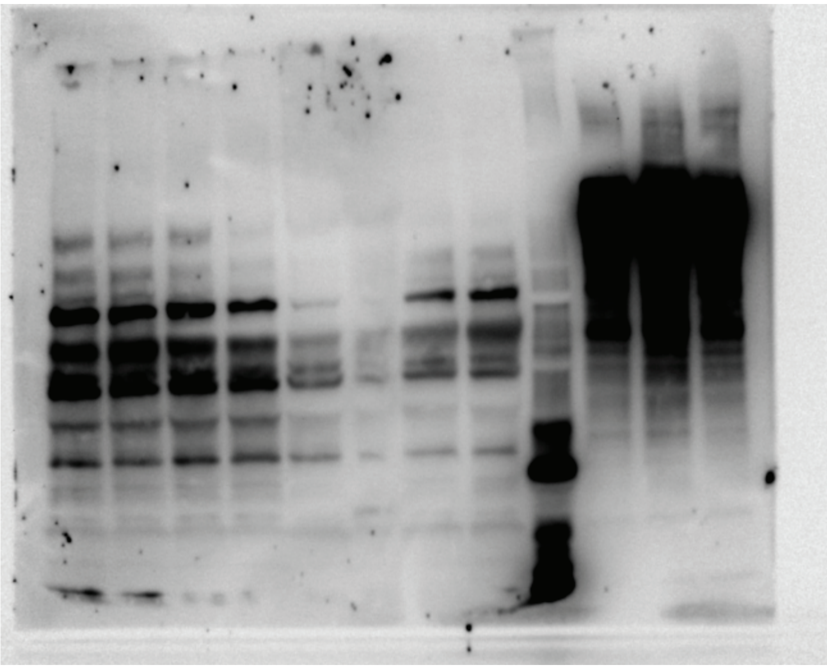

Anti-GAPDH

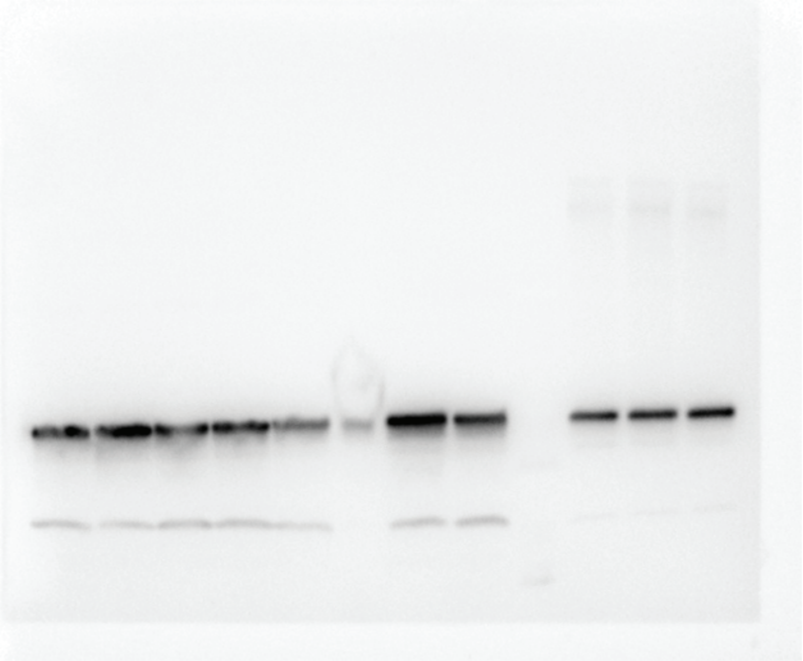

Precision Plus
Protein Dual
Color Standard

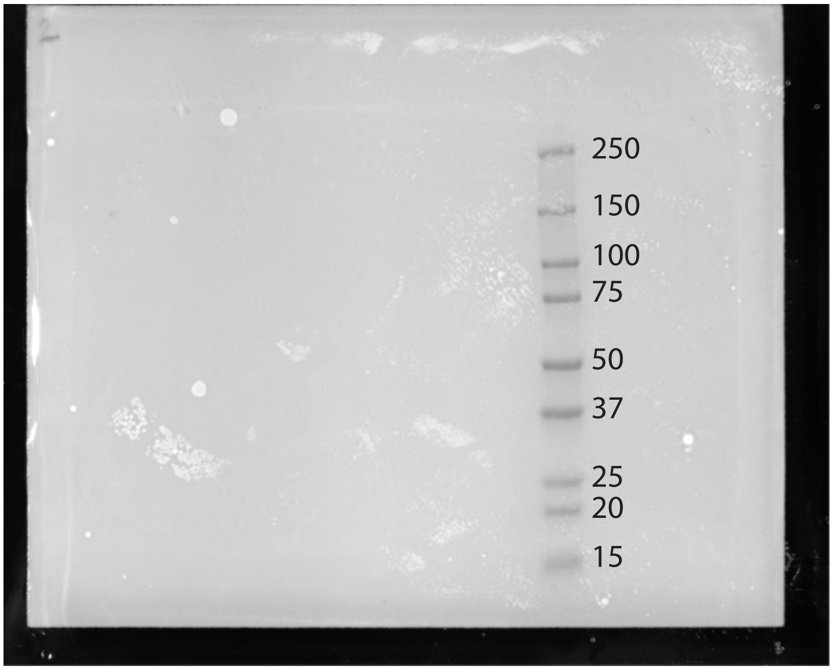

Lung

Anti-AAVR

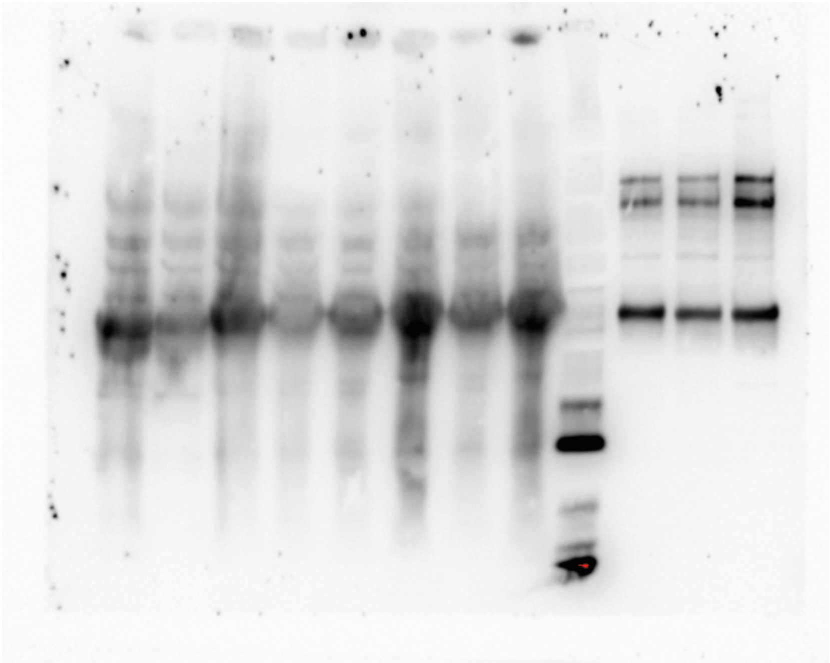

Anti-GAPDH

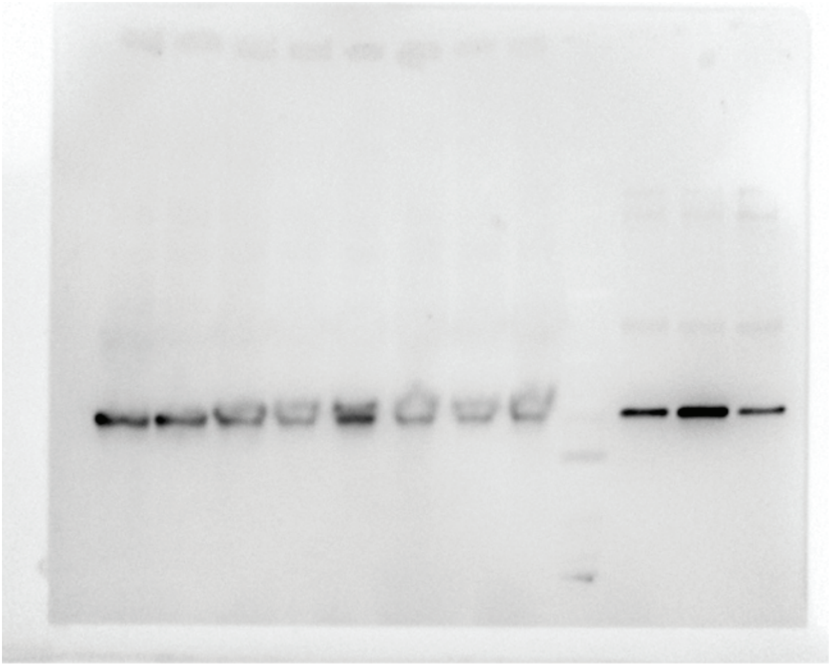

Precision Plus Protein Dual Color Standard

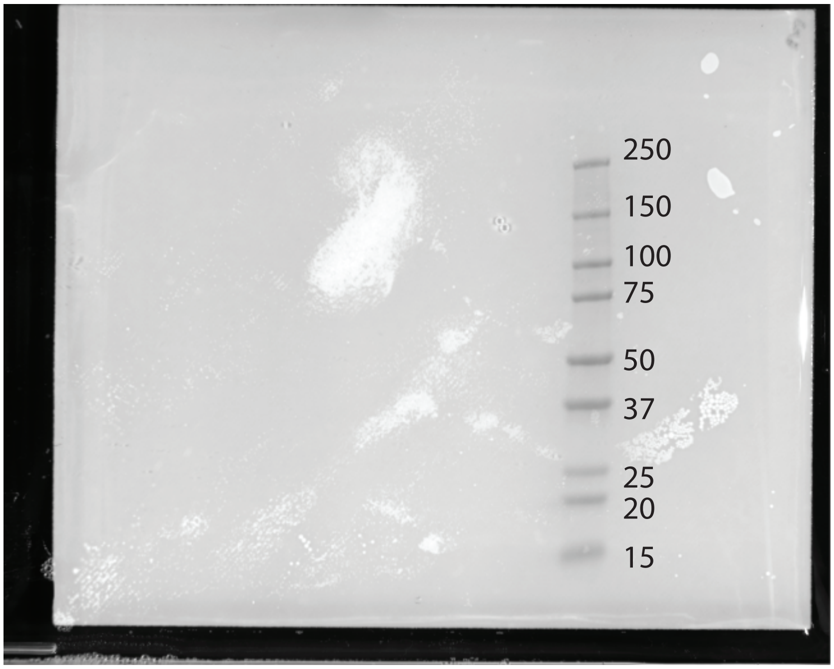

Eye

Anti-AAVR

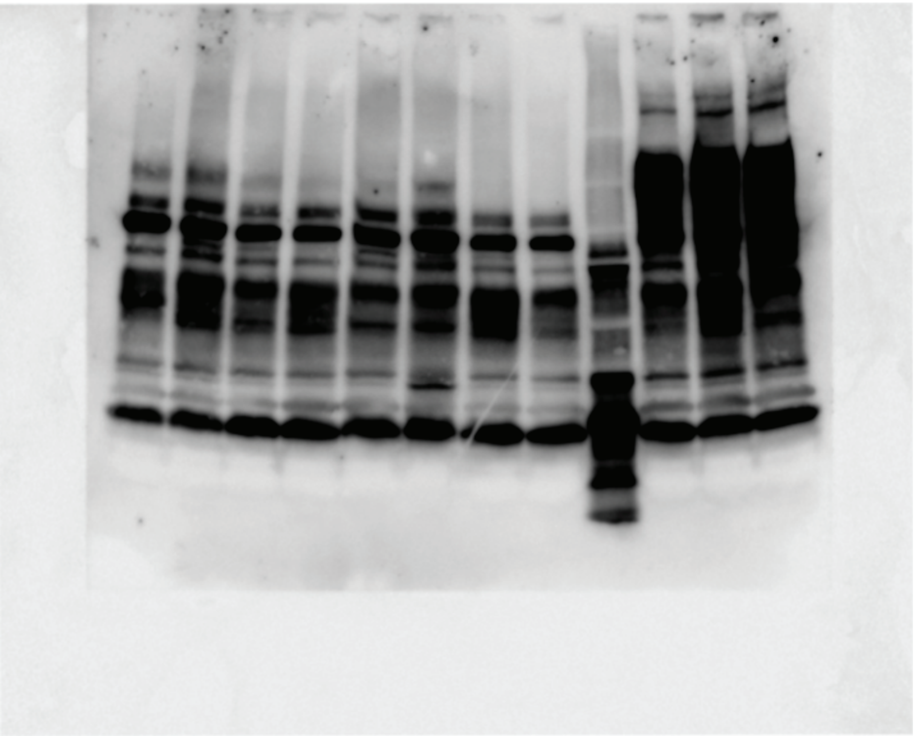

Anti-GAPDH

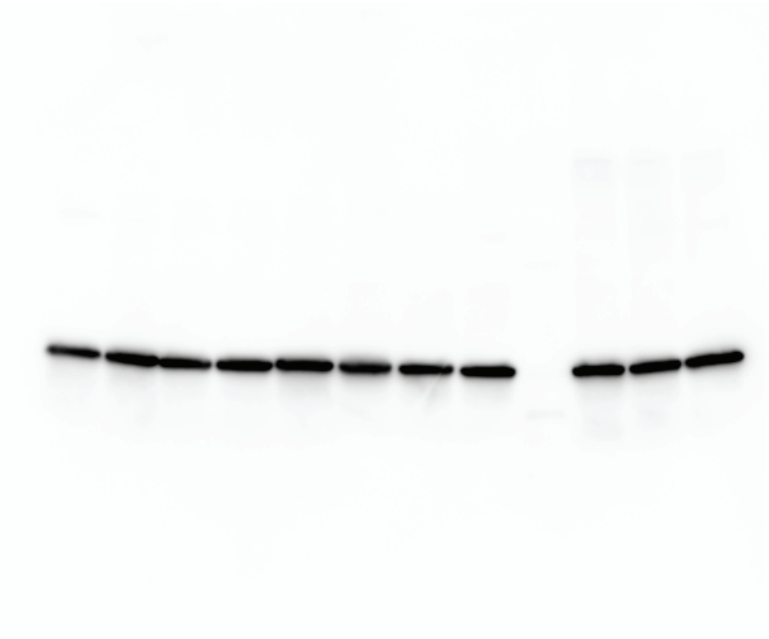

Precision Plus
Protein Dual
Color Standard

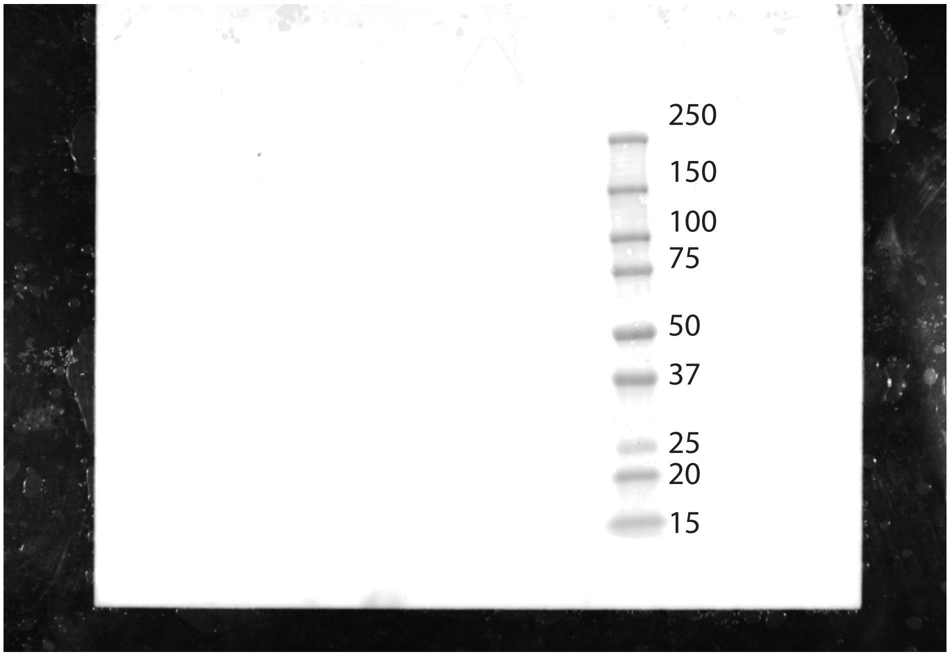

Muscle

Anti-AAVR

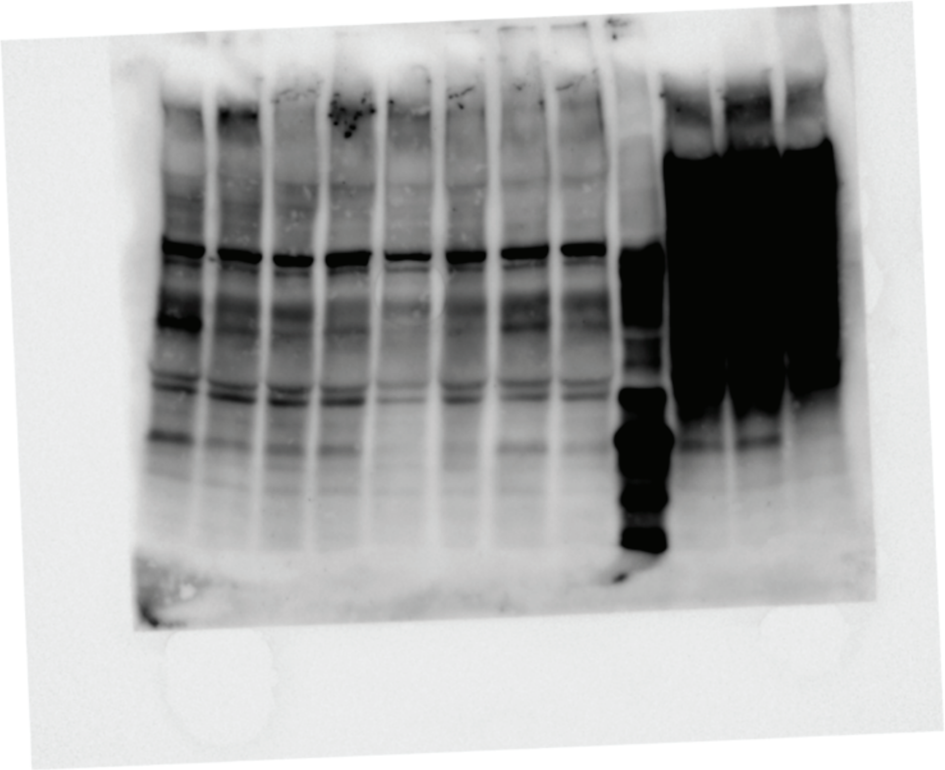

Anti-GAPDH

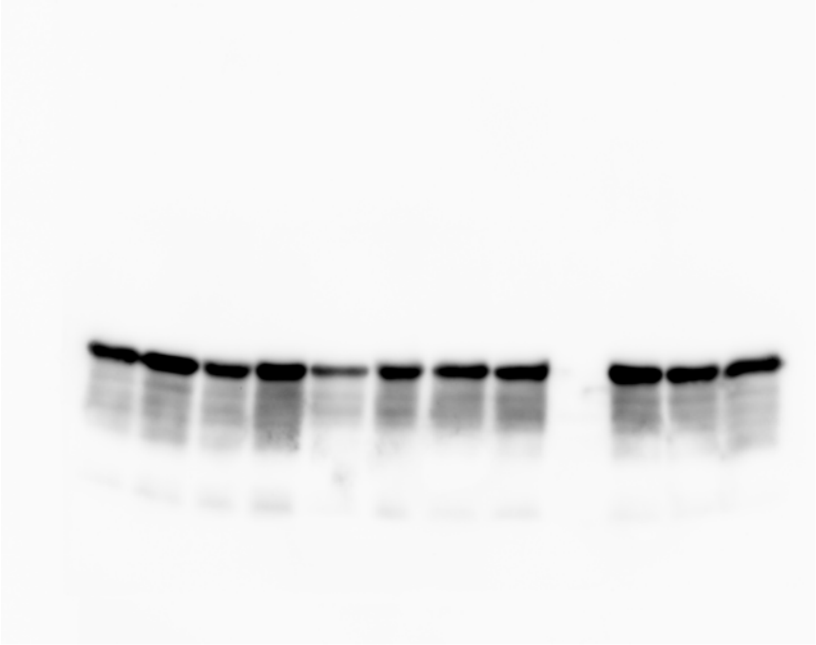

Precision Plus Protein Dual Color Standard

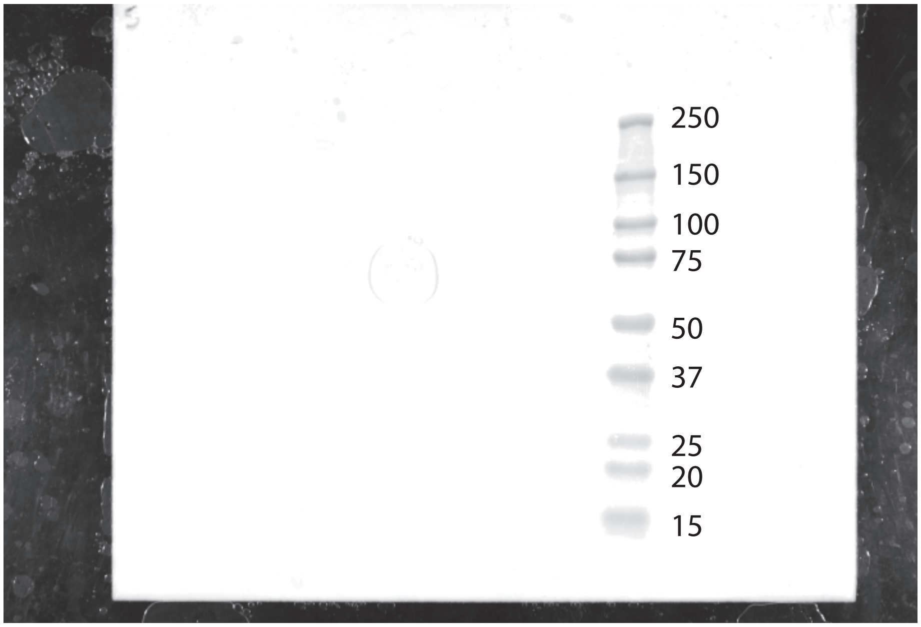

Kidney

Anti-AAVR

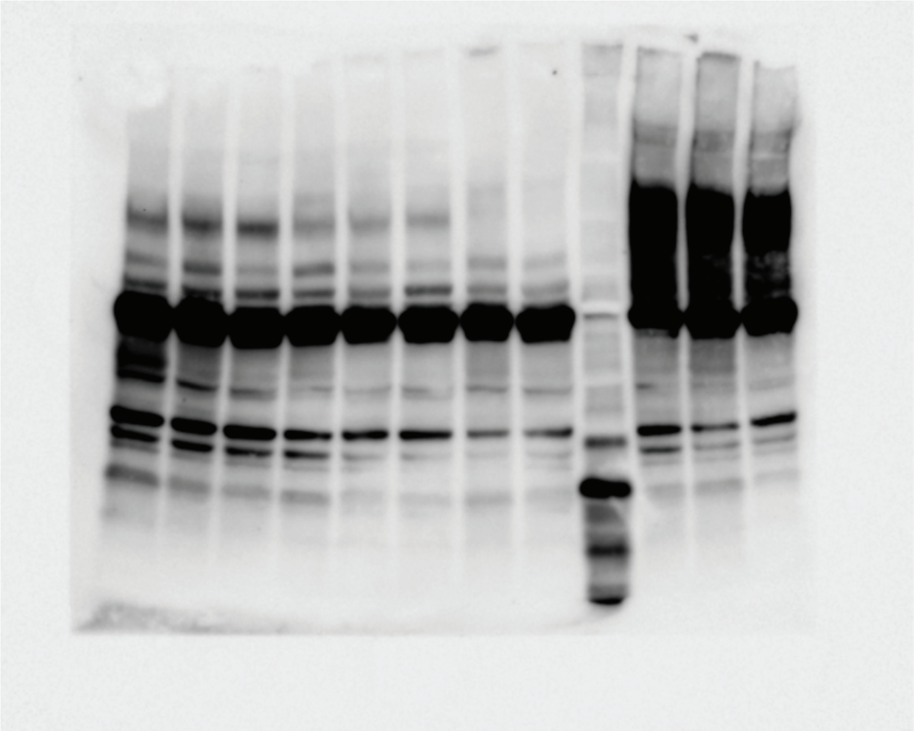

Anti-GAPDH

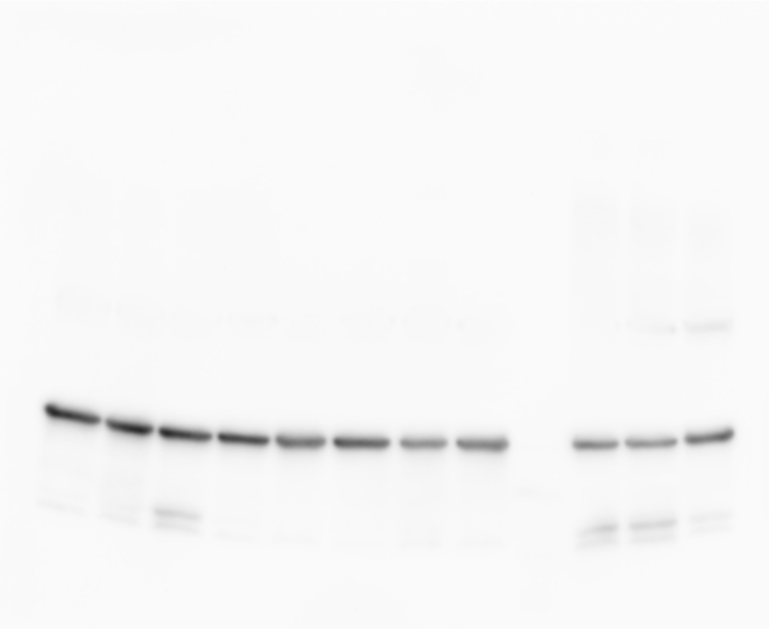

Precision Plus Protein Dual Color Standard

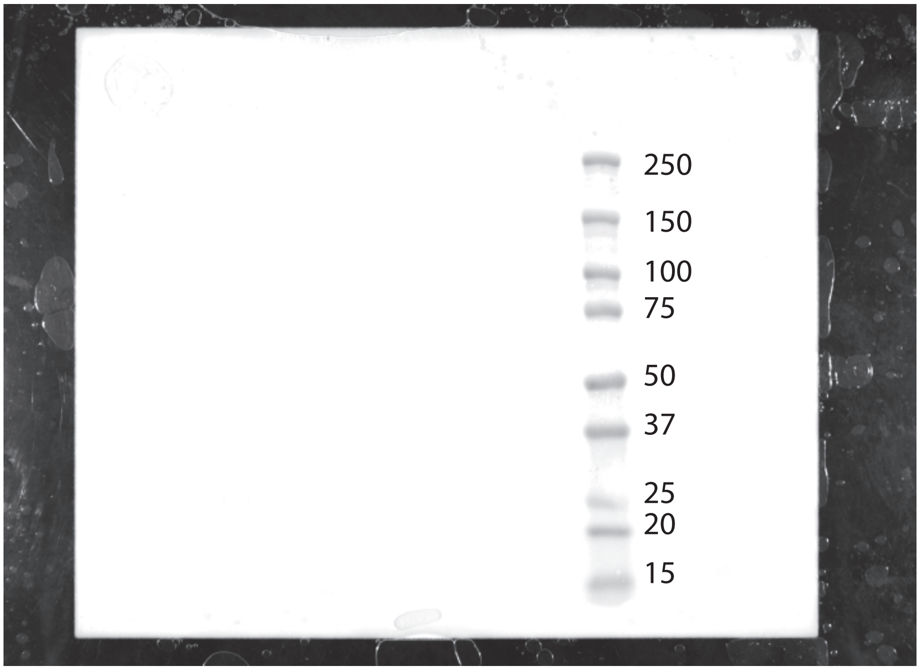

Brain

Anti-AAVR

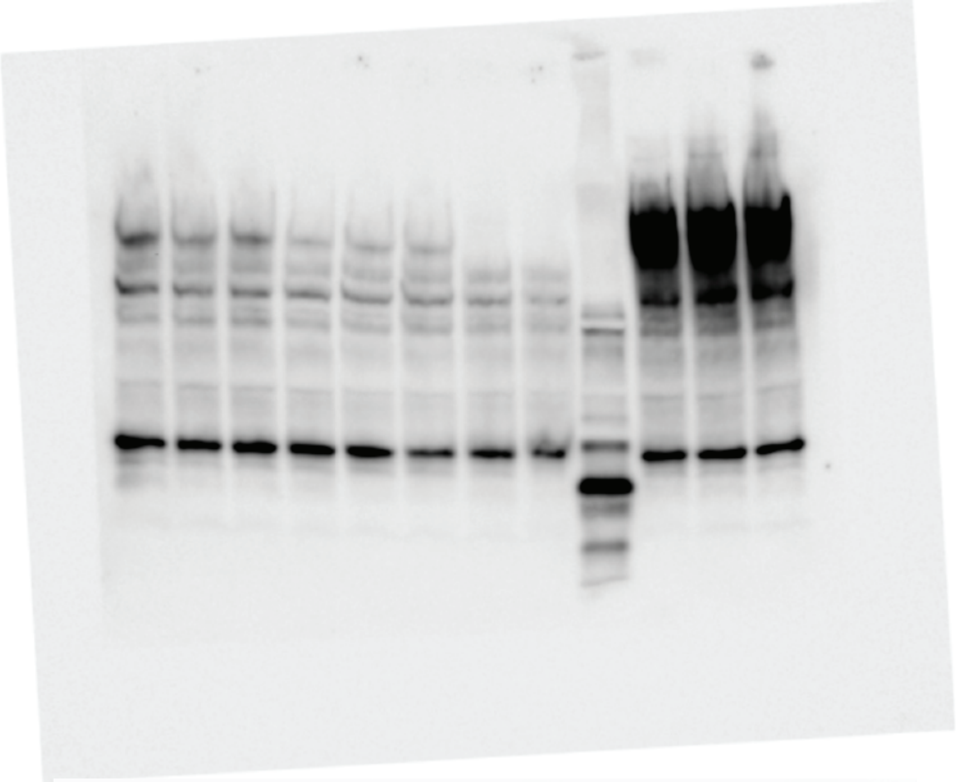

Anti-GAPDH

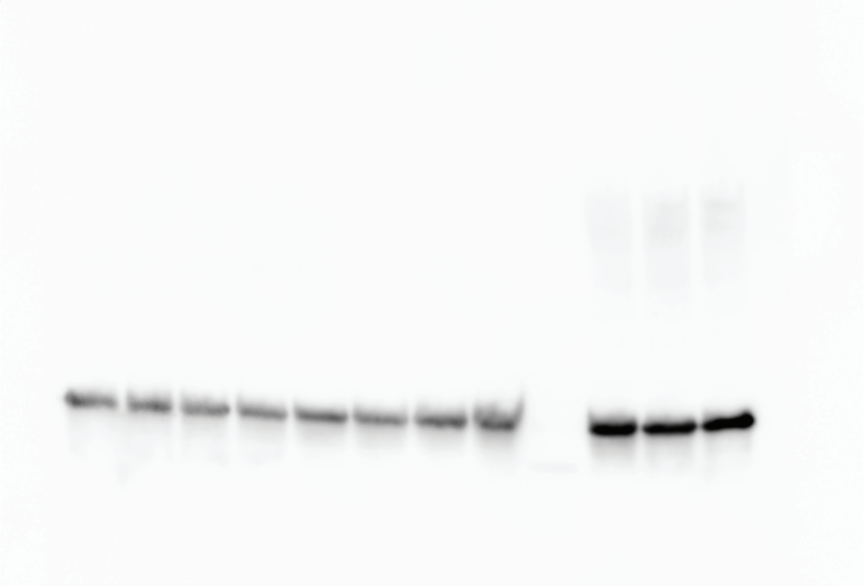

Precision Plus Protein Dual Color Standard

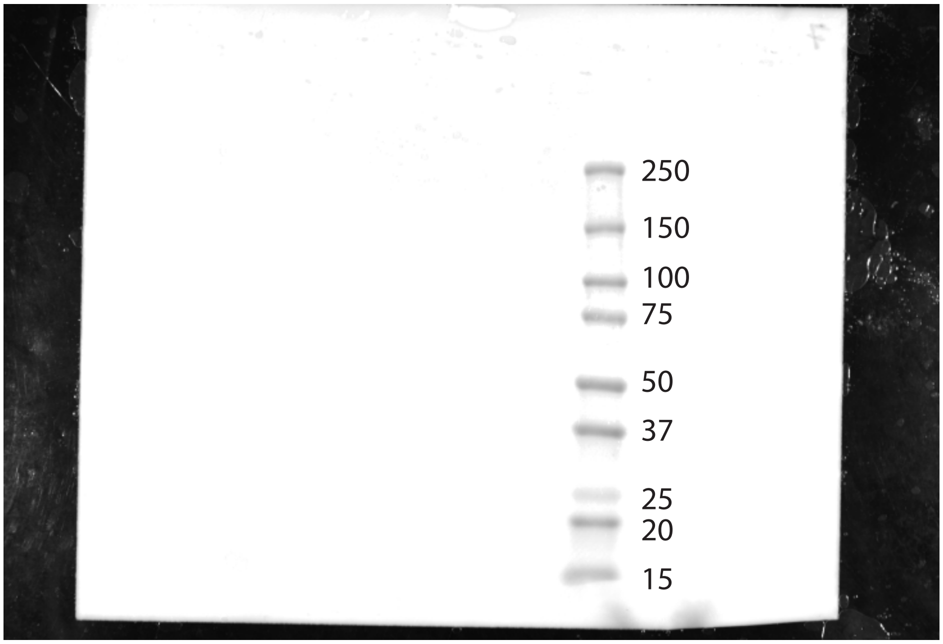

Supplement: Source Data Extended Data Fig. 2 — Full western blots. [file 41592_2023_1896_MOESM9_ESM.pdf]
